# Protein extraction from lupin (*Lupinus angustifolius* L.) using combined ultrasound and microwave techniques: Impact on protein recovery, structure, and functional properties

**DOI:** 10.1016/j.ultsonch.2025.107232

**Published:** 2025-01-13

**Authors:** Jiafei Tang, Lucie Cases, Sheila Alves, Da-Wen Sun, Brijesh K. Tiwari

**Affiliations:** a Teagasc Food Research Centre, Ashtown, Dublin, Ireland; b Food Refrigeration and Computerised Food Technology (FRCFT), School of Biosystems and Food Engineering, University College Dublin, National University of Ireland, Belfield, Dublin 4, Ireland; c Teagasc Crop Science Department, Oak Park, Carlow R93 XE12, Ireland

**Keywords:** Ultrasound, Microwave, Lupin protein extraction, Protein recovery rate, Structure properties, Functional properties

## Abstract

This study presents the first application of combined ultrasound (US) and microwave (MW) techniques for the efficient extraction of lupin protein isolates (LPI) from Irish-grown narrow-leaved lupin grain, variety ‘PRIMADONNA’. This variety was chosen due to its suitability for growth in the Irish temperate climate, which may influence protein extraction characteristics. By employing these emerging techniques, this research demonstrates a potential approach for improving protein recovery rates as well as enhancing the structural and functional properties of LPI. US and MW treatments significantly outperformed conventional extraction (CE), with combined US and MW treatments showing synergistic effects that significantly enhanced extraction yield and protein recovery rates of LPI (P < 0.05) within a shorter processing time (10 min) compared to 1 h CE. SDS-PAGE analyses revealed that US and MW treatment preserved the primary structure of LPI, while Fourier transform infrared spectrometer (FTIR) analyses showed that high-power MW induced significant changes in the secondary structure, transferring protein structures from highly ordered (β-sheet and α-helix) to disordered forms (random coil and β-turn). Lower power combinations (US50 + MW25) effectively improved protein solubility and reduced particle size, whereas higher power combinations (US50 + MW50, US100 + MW50) decreased solubility and increased particle size and viscosity significantly (p < 0.05). These findings underscore the potential of US and MW combinations for efficient lupin protein extraction, providing a new approach to sustainable plant extraction.

## 1 Introduction

The growing consumer demand for plant-based proteins as alternatives to animal proteins has sparked significant interest in the food industry. This shift is driven by increasing awareness of sustainability, health benefits, and ethical considerations [1]. Among various plant-based protein sources, lupin (*Lupinus* L., family Fabaceae) stands out as a promising candidate due to its high nutritional value, non-GMO status in commercial cultivation, superior sustainability, and lower production costs compared to conventional protein sources [1]. Shelled lupin seeds contain 39 %-53 % protein on a dry matter basis, significantly higher than soy, which contains less than 25 % protein [2]. Additionally, lupin consumption offers numerous health benefits, such as improved gut health and lower cholesterol levels, attributable to its high fibre and low starch content [3].

Traditional approaches for extracting protein from lupin involve methods such as alkaline extraction combined with isoelectric precipitation or ultrafiltration, salt-induced micellization with subsequent dilution precipitation, and acid extraction [2], [4]. Due to operational simplicity and cost considerations, alkaline extraction combined with isoelectric precipitation is the most commonly used technique. This technique employs an alkaline solution, maintaining a pH range of 8 to 9, to extract proteins, followed by isoelectric precipitation at a pH range of 4.5 to 5 for protein refinement [5], [6], [7]. To evaluate the structural and functional properties of extracted lupin proteins, several analytical techniques are crucial. Sodium dodecyl sulfate–polyacrylamide gel electrophoresis (SDS-PAGE) is widely used to determine the primary structure of proteins [8], which is essential to understanding whether the extraction process affects protein integrity. Fourier-transform infrared spectroscopy (FTIR) is critical for assessing secondary structural changes, such as the transition from α-helix and β-sheet to random coil structures, which can influence the protein's functionality and bioactivity [9]. Additionally, Protein solubility is a crucial factor in food science, significantly influencing the utilization of proteins as functional ingredients in food development and production by affecting the physicochemical properties, processing, sensory characteristics, shelf life, and nutritional value of protein-rich foods [10]. Particle size analysis directly affects texture and other functional properties in food systems while viscosity analysis could assess rheological properties of proteins, which is critical for various industrial applications, especially in food formulations where texture plays a significant role [11].

The growing demand for sustainable and energy-efficient methods has led to the development of innovative technologies such as ultrasound and microwave-assisted extraction [12], [13], [14], [15]. Ultrasound-assisted protein extraction relies on cavitation, where the pressure changes from sound waves create and rupture bubbles, releasing high energy and producing extreme temperatures (2000–5000 K) and pressures (300–1200 bar), effectively destroying plant cell walls and promoting the release of biologically active compounds into the solvent [16], [17]. Moreover, ultrasound (US) has been shown to modify the physical and chemical properties of proteins, affecting their structure and bioactive functionalities [18]. The cavitation effect produces free radicals that can alter the protein structures through oxidative reactions, leading to changes in bioactivity, which is essential in improving the functional properties of extracted proteins. For instance, US has been widely applied in the herbal industry, where it has been shown to enhance the bioactivity of herbal proteins through the disruption of covalent bonds and structural modifications [19].

Microwave-assisted extraction, on the other hand, is a green and novel method characterized by high reproducibility, efficiency, low energy consumption, and reduced solvent use [20]. Microwave (MW) technology disrupts hydrogen bonds in plant cell walls through dipole rotation and ion conduction, enhancing cell membrane fluidity and porosity [21]. This allows for better solvent penetration and more efficient release of intracellular compounds. Compared to traditional thermal treatments, MW can generate high thermal energy quickly, minimizing adverse effects on nutritional value and flavour [16]. It has been reported that MW has been studied for protein extraction from coffee green beans, peanut flour, coffee silverskin, and soymilk [12], [22], [23], [24]. In addition to increasing protein yield, MW treatment has also been reported to show a tendency to increase β-sheets and decrease α-helices, thereby altering the functional properties of the protein [22].

However, each technique has limitations when used alone. For instance, US may not completely disrupt plant cell structures, while MW treatment alone may not ensure uniform heating. Combining these techniques, known as ultrasound-microwave synergistic extraction (UMSE), has been shown to overcome these limitations by integrating the beneficial properties of both methods. UMSE uses the high energy effects of MW and ultrasonic cavitation to ensure uniform mixing and enhance the mass transfer of soluble components. Simultaneous UMSE can limit the inconsistent heating and minimal thermal effects observed in individual MW or US treatments [21]. Furthermore, the mechanical oscillation from ultrasonic cavitation can improve MW heating efficiency, while the thermal energy from MW can enhance the effects of US [25].

UMSE has been reported to have some applications in the extraction of various plant proteins, although it remains in its early stages for extracting proteins from plant materials. For example, Cheng et al. [26] enhanced the protein extraction procedure of *Moringa oleifera* leaves using response surface methodology, reporting that optimal extraction conditions are a solvent-to-solid ratio of 91:1 (v/w), an extraction time of 148 s, an extraction temperature of 41 °C, and a microwave power of 81 W, resulted in a protein yield of 82.07 mg/g, significantly higher than traditional solvent extraction. Similarly, Guan et al. [27] studied barley hordein extraction and demonstrated that UMSE significantly improved the extraction efficiency and reduced the extraction duration. Other studies include the extraction of proteins from pumpkin seeds [28], *Eurycoma longifolia* roots [29], and sweet potato [30] have also been reported to show the benefits of UMSE.

Despite the clear advantages of UMSE in extracting various plant proteins, its application in lupin protein extraction remains unexplored. This study aims to fill this gap by investigating the combined use of US and MW technologies in lupin protein extraction, focusing on the recovery rate, structural changes, and functional properties of the protein. By integrating these emerging technologies, this study offers a comprehensive evaluation of their potential impact on lupin protein extraction.

## 2 Materials and methods

### 2.1 Lupin cultivation

Certified seed from a single lupin (*Lupinus angustifolius* L.), variety ‘PRIMADONNA’, was sourced from Nordic Seeds (Galten, Denmark). The seeds, previously coated with lupins-specific rhizobium (LegumeFix®, UK), were sown on the 5th of April 2022, at Knockbeg (Teagasc experimental research farm) in Co. Laois, Ireland (GPS coordinates: 52°51′54.6″N, 6°56′32.8″W). A conventional one-pass system (Winter Steiger) was used, after ploughing, with a row space of 157 mm and a seed rate of 105 seeds/m^2^, for a total cultivated area of 240 m^2^. The lupin plants reached maturity and ripped in the field, and the crop was harvested on the 19th of August 2022, using a combine harvester, with a recorded yield of 5.12 t/ha, at 14 % moisture. Characteristics of sown seed and harvested grain are presented in Table 1.

**Table 1:** Characterization and quality of sown seed and harvested grain.

	Purity (%)	Germination (%)	TGW (g)	Moisture (%)	Protein (%)
Sown seed	99*	95*	170*	−	−
Harvested grain	99	−	142 ± 3	8 ± 1	31.8 ± 0.2

Note: TGW = thousand grain weight (the weight of 1000 seeds). Thousand grain weight was determined using a TSW & SEED BIOMETRY instrument (OPTO-AGRI). Protein (%) and moisture (%) were determined using an INFRATEC 1241 Grain Analyser (FOSS). * Value provided by supplier. − indicated the information was unavailable.

### 2.2 Materials and chemicals

Harvested lupin grains were stored in cool, dry conditions until needed. They were then milled into a fine powder using a Robot Mixer (43001R, Ireland). We maintained the samples at 4 °C before subsequent processing. Analytical-grade reagents were sourced from Sigma, Ireland, and distilled water was utilized for all procedures related to protein extraction and analysis.

### 2.3 Lupin protein extraction process

**Fig. 1:**
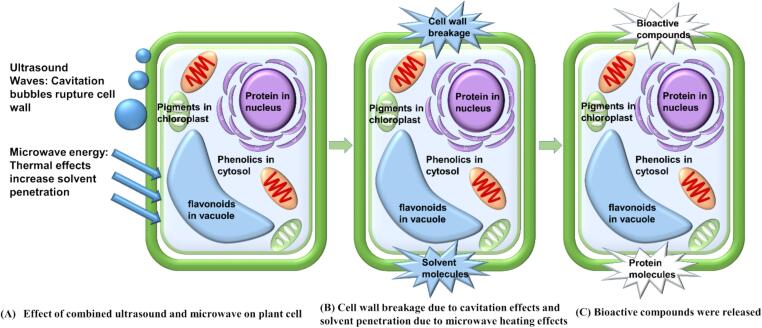
The combined mechanism of ultrasound and microwave extraction for plant proteins, which shows how ultrasound creates cavitation bubbles that rupture plant cell walls, while microwaves rapidly heat the material to enhance solvent penetration, and the figure was modified from Tang et al. [17].

In this study, an ultrasound-microwave-assisted extractor, model E200, produced by Idco SAS (Marseille, France) was used. The E200 includes separate ultrasound control (0–200 W) and separate microwave control (200–2000 W), equipped with a sample tank, allowing the concurrent use of microwave and ultrasound techniques. Fig. 1 illustrates the combined mechanism of ultrasound and microwave extraction for plant proteins. Ultrasound generates cavitation bubbles that rupture the plant cell walls, while microwaves rapidly heat the material, enhancing solvent penetration. The synergistic effect of these two techniques significantly improves the efficiency of protein extraction.

**Fig. 2:**
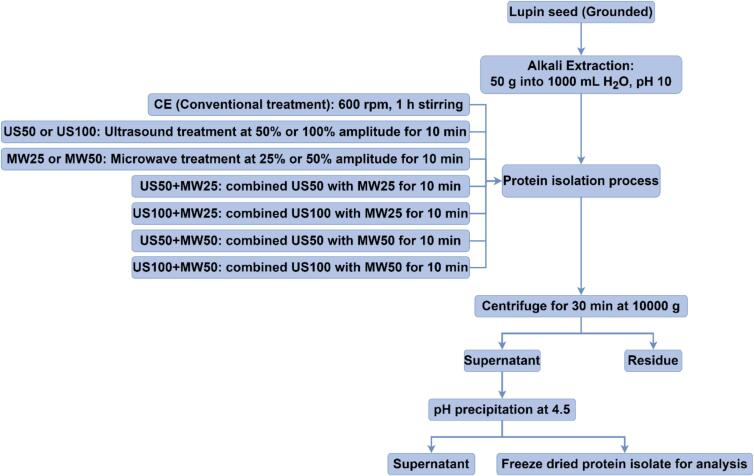
The flowchart of lupin protein extraction.

The experiment flowchat, illustrated in Fig. 2, involved different treatments: conventional extraction (CE), ultrasound treatment with 50 % or 100 % amplitude (US50 or US100), microwave treatment with 25 % or 50 % amplitude (MW25 or MW50), and combined treatments, including US50 + MW25, US100 + MW25, US50 + MW50, and US100 + MW50, were performed at the laboratory scale with 50 g of lupin flour. The proximate composition of the lupin flour is detailed in Table 2. For the extraction procedure, 50 g of lupin flour was mixed with 1000 mL of distilled water at a ratio of 1:20 g/mL. The pH of the suspension was modified to 10 using a 1 mol/L NaOH solution.

**Table 2:** Proximate composition of dry lupin flour.

Components	Content (%)
Protein	30.05 ± 0.06
Carbohydrate	54.99 ± 0.05
Moisture	7.56 ± 0.03
Lipid	5.02 ± 0.01
Ash	2.38 ± 0.06

Note: Results are presented as mean ± SD.

CE was conducted as a control treatment, modified from the method detailed by Tanger et al. [31]. This involved placing the suspension in a Firex crucifix mixer (Sedico, Italy) stirring at 600 rpm for 1 h. Other treatments using E200, including US50, US100, MW25, MW50, US50 + MW25, US100 + MW25, US50 + MW50, and US100 + MW50, were performed after pouring the 1000 mL sample suspension into the tank, treating with corresponding US and MW parameters for a period of 10 min.

### 2.4 Lupin protein recovery process

After the protein was extracted into the suspension, the supernatant was obtained by centrifuging the mixture at 10,000 g for 20 min, which was adjusted to pH 4.5, the isoelectric point, using 1 M HCl to precipitate the protein. This precipitated protein was further separated by centrifugation and then freeze-dried to produce lupin protein isolates (LPI). The nitrogen content of the LPI was conducted with an FP-328 nitrogen analyser (Leco Corporation, USA), using a conversion factor of 6.25 [32]. We determined the yield and recovery rate of the LPI protein extraction using the formulas below:

(1) $$Protein\;recovery\;rate\;\left(\%\right)=\frac{Protein\;content\;in\;dried\;precipitate\;collected\;\left(g\right)}{Protein\;content\;in\;lupin\;flour\;samples\;\left(g\right)}\times 100$$

(2) $$Protein\;yield\;\left(\%\right)=\frac{Quantity\;of\;dried\;precipitate\;collected\;\left(g\right)}{Quantity\;of\;lupin\;flour\;samples\;\;\left(g\right)}\times 100$$

### 2.5 Structural and functional attributes of LPI

#### 2.5.1 Primary structure: SDS-PAGE analysis

The protein primary structure of LPI was evaluated using SDS-PAGE, with the modifications detailed by Santos-Hernández et al. [33]. According to the protein content, approximately 4.09 to 5.31 mg of LPI samples were weighed and mixed with equal parts of distilled water and Laemmli 2 × Concentrate to achieve about 4 mg/mL of protein concentration. These mixtures were heated at 90 °C for 15 min to dissolve the protein. After centrifugation, 15 μL supernatant was placed into a 4–20 % Criterion™ TGX Stain-Free™ Protein Gels (Bio-Rad) using a Tris/Glycine Buffer. Electrophoresis was conducted at 5 W for 1.5 h. The gels were treated with Coomassie Brilliant Blue R-250 for staining. The images were taken with a GS-800 Calibrated Densitometer (Bio-Rad, Germany).

#### 2.5.2 Secondary structure: FTIR analysis

The FTIR of LPI was obtained by an FTIR spectrometer (Thermo Scientific, USA), as decribed by Tang et al. [18]. The single-beam reflectance spectra obtained were transformed to absorbance spectra within the 450–4000 cm^−1^ wavelength range, with a resolution of 2 cm^−1^. Prior to each measurement, an air-blank background calibration was performed. To ensure accuracy, we performed 64 scans for each measurement to average the spectral data. The acquisition process was managed with OMNIC software (Thermo Fisher Scientific, USA). All samples underwent quadruplicate measurements.

#### 2.5.3 Protein solubility analysis

Protein solubility of LPI was conducted with a method described by Bradford [34], at 5 different pH levels. LPI suspensions for all samples were made by dispersing LPI in distilled water (1 % g/mL) and 0.1 M NaOH or 0.1 M HCl was used to adjust their pH at the levels of 2.5, 4.5, 6, 8 and 10. Then the suspension was stirred vigorously for 1 h and centrifuged at 11000 rpm for 10 min at 20 °C. An amount of 10 μL of the sample was added into the 96-Well microplate (Thermo Scientific) and 300 μL of the Pierce™ Bradford Plus Protein Assay Reagent was placed into each well and then mixed for 30 S. After 10 min incubation, the UV VIS spectrophotometer (Perkin Elmer-Lambda 35, USA) was used to measure the samples’ absorbance at 595 nm. All measurements were conducted in duplicate. BSA standards from the Pierce™ Bradford Plus Protein Assay kit provided were used to establish the standard curve. We calculated protein solubility using the following method [35]:

(3) $$Solubility\;of\;LPI\;\left(\%\right)=\frac{Protein\;content\;in\;LPI\;supernatant\;}{Protein\;content\;in\;LPI\;sample\;}\times 100$$

#### 2.5.4 Protein particle size analysis

We characterized the particle size distribution of LPI using static laser light diffraction (Mastersizer 2000, UK) [36]. LPI suspensions for all samples were prepared by dispersing LPI in distilled water (1 % g/mL) and adjusted at pH 7 with phosphate-buffered saline (PBS). LPI suspension was equilibrated at room temperature (about 25 °C) and placed into a dispersion unit, where distilled water served as the dispersant agent. The settings for lupin protein included a refractive index of 1.45, an absorption index of 0.1, and a dispersant refractive index of 1.33. The results were recorded until the laser obscuration approached 12 %. The particle size median (D50) was used to represent the results.

#### 2.5.5 Rheological properties

To evaluate the rheological characteristics of LPI, an Anton Paar MCR301 controlled stress rheometer (Anton Paar GmbH, Austria), equipped with a 25 mm serrated parallel plate and base plate, was used to measure viscosity and shear rate. The LPI suspensions (15 % g/mL) were prepared by dispersing LPI in distilled water and adjusted at pH 7 with PBS buffer. 1 mL of the resulting suspension was placed on the rheometer, after resting for 10 min at 25 °C. The test was conducted with a frequency range of 0.01 to 10 Hz, a constant shear strain of 0.01 %, a gap of 1 mm, and a waiting time of 5 min. An amplitude sweep (ranging from 0.001 % to 0.05 %) was performed initially to evaluate the linear viscoelastic region of LPI.

### 2.6 Data analysis

The data were represented as mean ± standard deviation (SD). The differences between various treatments were analysed by one-way ANOVA and followed up with Tukey’s HSD test for multiple comparisons. Statistical significance was determined at p < 0.05.

## 3 Results and discussion

### 3.1 Protein recovery efficiency

**Fig. 3:**
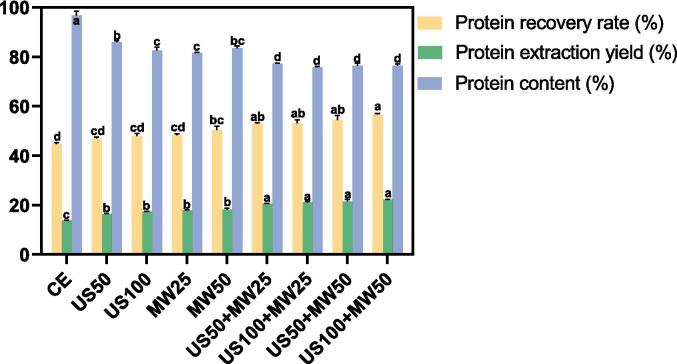
The influence of different treatments on the recovery of lupin protein. Bars with different letters within various treatments indicate significantly differences (p < 0.05).

Alkaline pH solvents are often employed for extracting legume proteins, and the concentrated protein isolate could be obtained by adjusting pH to the protein isoelectric point. Table 3 and Fig. 3 show the LPI protein content, extraction yield, and recovery rates obtained from different treatments.

**Table 3:** The influence of combined US and MW techniques on the recovery of lupin protein.

Technique	Protein content (%)	Extraction yield (%)	Protein recovery rate (%)
US100 + MW50	76.40 ± 0.64^d^	22.30 ± 0.03^a^	56.69 ± 0.41^a^
US50 + MW50	76.46 ± 0.99^d^	21.39 ± 0.98^a^	54.41 ± 1.78^a,b^
US100 + MW25	75.70 ± 0.45^d^	21.07 ± 0.41^a^	53.08 ± 1.35^a,b^
US50 + MW25	77.21 ± 0.26^d^	20.61 ± 0.07^a^	52.95 ± 0.36^a,b^
MW50	83.57 ± 0.71^b,c^	18.12 ± 0.71^b^	50.38 ± 1.54^b,c^
MW25	81.50 ± 0.25^c^	17.82 ± 0.20^b^	48.33 ± 0.39^c,d^
US100	82.50 ± 1.36^c^	17.43 ± 0.10^b^	47.85 ± 1.06^c,d^
US50	85.97 ± 0.42^b^	16.41 ± 0.27^b^	46.95 ± 0.54^c,d^
CE	96.76 ± 1.63^a^	13.89 ± 0.07^c^	44.72 ± 0.52^d^

Note: Data are presented as the mean ± SD from two replicates. Different letters (a, b) within the same column indicate significant differences (p < 0.05). US100 + MW50: combined 100 % amplitude ultrasound with 50 % amplitude microwave, US50 + MW50: combined 50 % amplitude ultrasound with 50 % amplitude microwave, US100 + MW25: combined 100 % amplitude ultrasound with 25 % amplitude microwave, US50 + MW25: combined 50 % amplitude ultrasound with 25 % amplitude microwave, MW50: 50 % amplitude microwave, MW25: 25 % amplitude microwave, US100: 100 % amplitude ultrasound, US50: 50 % amplitude ultrasound, and CE: Conventional method.

Ultrasound (US) treatment alone was conducted at 50 % (100 W) and 100 % (200 W) ultrasonic amplitudes for 10 min, resulting in extraction yields of 16.41 ± 0.27 % and 17.43 ± 0.10 %, and protein recovery rates of 46.95 ± 0.54 % and 47.85 ± 1.06 %, respectively. It was observed that with an increase in US amplitude, the extraction yield and protein recovery rate of LPI slightly increased, despite the difference not being statistically significant (P > 0.05). However, both ultrasonic amplitudes improved protein recovery rates and significantly increased protein extraction yields compared to conventional extraction (CE) over 1 h (P < 0.05). The improvement is likely due to cavitation induced by ultrasound, which breaks and degrades the plant matrix, enhancing the solution's penetration into the internal structure, thus improving protein extraction efficiency [17], [37]. Similar findings were reported in previous studies, for example, Aguilar-Acosta et al. [38] reported that ultrasonic treatment was beneficial to improve the protein yield of lupin (*L. mutabilis*). Despite the increase in extraction yield, the protein content of LPI decreased after US treatment compared to CE, with US50 and US100 producing protein contents of 85.97 ± 0.42 % and 82.50 ± 1.36 %, respectively, which were significantly lower than the 96.76 ± 1.63 % observed in CE. This decrease may be attributed to the fact that ultrasound not only enhances protein release but also facilitates the extraction of other bioactive compounds, such as polysaccharides or phenolics, which dilute the protein concentration in the final isolate.

Microwave (MW) treatment alone was performed at amplitudes of 25 % (500 W) and 50 % (1000 W) for 10 min. Consistent with US treatment results, the extraction yields (17.82 ± 0.20 % and 18.12 ± 0.71 %, respectively) and protein recovery rates (48.33 ± 0.39 % and 50.38 ± 1.54 %, respectively) for both MW treatments were significantly higher than those of the CE group (1 h) (P < 0.05), and they increased with amplitude, albeit not significantly. However, MW treatment also resulted in a lower protein content compared to CE. The protein content was 83.57 ± 0.71 % and 81.50 ± 0.25 % for MW50 and MW25, respectively, which were still lower than the CE group. Although no literature was found that specifically addresses the effect of MW on lupin protein extraction, studies showed that MW-assisted extraction at 725 W for 8 min significantly improved peanut protein extraction yields (P < 0.05) [39]. Furthermore, Wen et al. [12] reported that MW extraction of coffee silverskin (CSS) could increase the protein recovery rate by 5.8 to 7 times compared to CE. The enhancement in protein recovery by MW treatment may be due to MW radiation directly heating the plant matrix's interior, increasing local temperature and pressure, thereby enhancing the transfer of target compounds into the solvent [12], [40].

After examining the effects of US and MW treatments alone, lupin was treated with combinations of US (50 % and 100 % amplitude, respectively) and MW (25 % and 50 % amplitude, respectively) to study their combined effects. The results indicated that extraction yield and protein recovery rate from LPI improved with increasing amplitude, although there was no significant difference among the four combined treatments. Additionally, extraction yields and protein recovery rates in all combined treatments were significantly better than those using US or MW alone (P < 0.05), suggesting a synergistic effect in lupin protein extraction. This synergistic effect might be due to the mechanical oscillation and stirring caused by ultrasonic cavitation, leading to uniform microwave heating and thereby improving extraction efficiency [21]. However, Ochoa-Rivas et al. [39] reported no synergistic effect in peanut protein extraction with sequential US-MW extraction, unlike our simultaneous application of both techniques in the same reactor. Similarly, the protein content in the combined US-MW treatments remained lower than in the CE group. For example, US100 + MW50 resulted in a protein content of 76.40 ± 0.64 %, which is significantly lower than the 96.76 ± 1.63 % observed in CE. This reduction in protein content could again be due to the simultaneous release of other bioactive compounds alongside the proteins, leading to a dilution effect in the final isolate.

### 3.2 Protein primary structure changes

Lupins are abundant in diverse proteins, primarily including four types of globulins: α-, β-, γ-, and δ-conglutins [41]. β-conglutin (7S) has a trimeric quaternary structure composed of 10–12 major subunits ranging from 15 to 72 kDa, while α-conglutin (11S) exhibits a hexameric structure, containing four major subunits (53, 60, 66, and 70 kDa), both of which are major storage proteins [42]. δ-conglutin (2S) is a monomeric protein comprising a small subunit and a large subunit linked by disulfide bonds, with a molecular weight typically below 20 kDa [1]. γ-conglutin (7S) is a minor component, composed of one major subunit (42–43 kDa) [42].

**Fig. 4:**
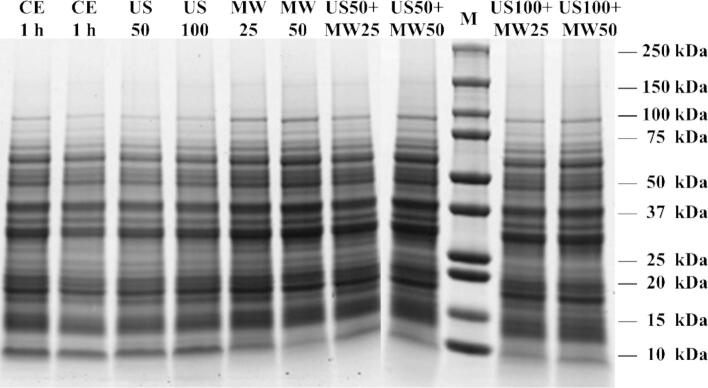
SDS-PAGE results of lupin proteins isolates under reducing conditions. Lane M indicates the standard protein marker.

Lupine protein profiles were conducted by SDS-PAGE, and Fig. 4 showed the protein bands with reducing conditions from various treatments. A highly abundant 19 to 60 kDa band was observed in SDS-PAGE across all treatment conditions, corresponding to non-covalently linked β-conglutin subunits [41]. Additionally, the 46 kDa and 36 kDa bands were prominent and may be related to the reduced α-conglutin subunits. Bands at 30 kDa and 17 kDa were also found in all protein extracts, potentially corresponding to γ-conglutin subunits. Under reducing conditions, the 43 kDa γ-conglutin was divided into two subunits of 30 and 17 kDa [41]. Bands below 17 kDa in SDS-PAGE were attributed to δ-conglutin. Shrestha et al. [1] reported that four δ-conglutin gene sequences (δ-1 to δ-4) with molecular weights of 17.8, 17.8, 17.4, and 10.7 KDa had been identified in *L. angustifolius*. Compared with CE and US treatments, the δ-conglutin bands (∼ 10 kDa) under other MW treatment conditions were significantly weaker, which might be attributed to reduced extraction of δ-conglutin due to MW-induced solvent boiling [20]. Furthermore, under MW treatment conditions, some large molecular weight (∼ 98 kDa) protein bands intensified, possibly due to protein aggregation at the submicron level.

### 3.3 Secondary structure changes of LPI protein

**Fig. 5:**
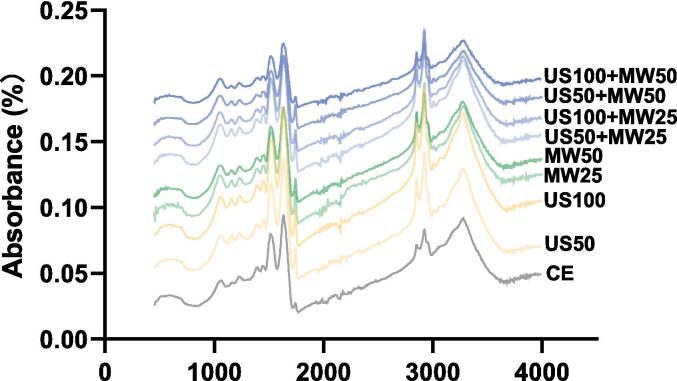
The Fourier transform infrared spectra of lupin protein isolates from different treatments.

FTIR results of lupin protein extracts subjected to different treatments provided a crucial evaluation of the induced structural changes. The 1600–1700 cm^−1^ range corresponds to the amide I band, which reflects changes in the secondary structure of lupin protein, including β-sheet, random coil, α-helix, and β-turn [43]. To better understand the effects of various treatments on the protein’s secondary structure, the obtained FTIR spectra were processed using second-order derivative analysis and Gaussian fitting (Fig. 5 and Table 4). The 1610–1640 cm^−1^ and 1673–1677 cm^−1^ correspond to β-sheet structures, 1641–1649 cm^−1^ correspond to random coil structures, and 1649–1657 cm^−1^ correspond to α-helix structures. In addition, β-turn structures are indicated by ranges of 1659–1674 cm^−1^ and 1681–1696 cm^−1^ [18].

**Table 4:** Secondary structure composition of LPI from various technologies.

**Technology**	**β-sheet/%**	**random coil/%**	**α-helix/%**	**β-turn/%**
CE	57.15 ± 0.28^a^	8.40 ± 0.39^c^	9.40 ± 0.15^a^	25.05 ± 0.26^c^
US50	49.70 ± 0.19^e^	7.87 ± 0.24^d^	9.29 ± 0.72^a^	33.14 ± 0.51^a^
US100	51.41 ± 0.29^d^	7.78 ± 0.42^d^	9.57 ± 0.09^a^	31.24 ± 0.81^a,b^
MW25	53.41 ± 0.38^c^	8.18 ± 0.94^c,d^	8.70 ± 0.23^b^	29.71 ± 0.61^b^
MW50	50.86 ± 0.61^d,e^	9.33 ± 0.58^a^	7.78 ± 0.51^c^	32.04 ± 0.08^a^
US50 + MW25	52.72 ± 0.23^c,d^	8.56 ± 0.13^c^	9.36 ± 0.25^a^	29.36 ± 0.83^b^
US100 + MW25	55.32 ± 0.34^b^	8.71 ± 0.78^b^	9.22 ± 0.38^a^	26.76 ± 0.37^b,c^
US50 + MW50	55.67 ± 0.61^b^	8.85 ± 0.20^b^	7.68 ± 0.20^c^	27.80 ± 0.36^b,c^
US100 + MW50	56.25 ± 1.01^a,b^	8.90 ± 0.87^b^	7.28 ± 0.41^c^	27.57 ± 0.52^b,c^

Note: Results are shown as mean ± SD of two replicates. Significant differences (p < 0.05) within the same column are indicated by different letters (a, b).

Peptide chains composed of random coils exhibit irregular and less stable structures, while α-helix represents highly organized and relatively stable secondary structures [44]. Cheng and Cui [45] reported that various hydrogen bonding patterns connect β-sheets, stabilizing these secondary structures. Compared to the CE method (1 h), US alone (10 min) including US50 and US100 resulted in fewer random coils and similar levels of α-helices, indicating that sonication does not significantly denature proteins. This outcome aligned with the findings of Ajayi et al. [46], who observed low protein denaturation efficiency with ultrasound when jack bean protein (JBP) was extracted using various mechanical methods, including thermal shearing, US, and MW treatment. However, microwave treatment significantly increased protein denaturation. When MW power was increased from 25 % (500 W) to 50 % (1000 W) for 10 min, the random coils and β-turns content showed a significant increase (P < 0.05), while the α-helices and β-sheets content showed a significant decrease (P < 0.05). This indicated that the thermal effects of MW denatured proteins disrupted hydrogen bonds, and transformed protein structures from highly ordered (β-sheet and α-helix) to disordered forms (random coil and β-turn). This trend was also observed in combined treatments. The random coil and α-helix contents of the US50 + MW25 and US100 + MW25 treatments were comparable to those of CE, but with increased MW power, the random coil content in the US50 + MW50 and US100 + MW50 treatments increased, and the α-helix content decreased (P < 0.05). Sun et al. [47] confirmed these findings, showing a reduction in ordered structures, including α-helix and β-sheet, and a rise in disordered structures (random coil) in microwave-treated pigeon peas (*Cajanus cajan*). Similarly, Ajayi et al. [46] found a higher proportion of disordered structures in microwave-treated JBP, attributed to the MW electromagnetic heating mechanism, which strongly disrupted protein interactions. Secondary structure changes of lupin proteins caused by MW and US treatments can also explain changes in the solubility and particle size of LPI.

### 3.4 Solubility analysis

**Fig. 6:**
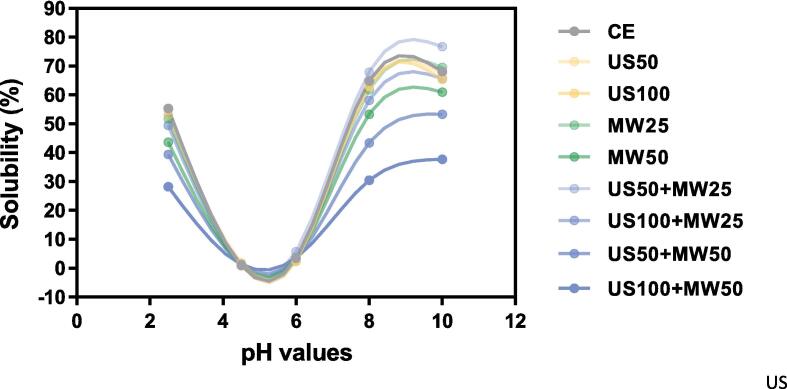
Solubility profile of lupin protein isolate (LPI) after E200 treatment with different parameters (US50, US100, MW25, MW50, US50 + MW25, US50 + MW50, US100 + MW25, and US100 + MW50) against the CE. Protein solubility has been measured at a range of pH from 2.5 to 10, at 25 °C.

Protein solubility is a significant factor, which is related to various functional characteristics like gelling, emulsification and foaming [1], [2]. Given its significance, identifying effective strategies to enhance the solubility of lupin protein is crucial for its broader application in commercial food products. The provided solubility curve graph (Fig. 6) demonstrated the impact of various E200 treatments on lupin protein isolate (LPI) solubility across a pH range of 2.5 to 10, in comparison to a control method (CE) involving 1 h of stirring. The treatments examined included 50 % and 100 % amplitude ultrasonic (US50 and US100) and 25 % and 50 % amplitude microwave (MW25 and MW50) treatments, alongside their combinations, each applied for 10 min.

Observations from Fig. 6 revealed a U-shaped solubility pattern, with the lowest solubility near the protein's isoelectric point (typically between pH 4.5 to 5.5) and higher solubility at lower and higher pH values. This trend aligned with results from Chew et al. [48], who found similar solubility patterns for LPI obtained through alkaline extraction followed by isoelectric precipitation and ultrafiltration across a pH range of 2–8, peaking at pH 2 and 8 while hitting a low between pH 4–5. Notably, 10 min of ultrasonic treatment alone (US50 and US100) yielded a solubility comparable to 1 h CE, indicating that ultrasonic treatment would effectively improve the solubility of LPI. This enhancement might be due to the disintegration effects of acoustic cavitation generated by ultrasonic waves, which fragment protein molecules into smaller aggregates, a phenomenon confirmed in previous research [11], [49], [50]. In addition, compared with CE, it could be found that the solubility of LPI in the US50 + MW25 treatment was significantly increased, suggesting that the synergistic application of ultrasound and microwave could serve as a potent method for solubility enhancement of LPI. The cavitation effect produced by US and the rapid heating of MW may disrupt hydrogen and hydrophobic bonds and expose hydrophilic groups of amino acids, thus reducing LPI's particle size and enhancing its water interaction surface area. This was supported by similar findings from Li et al. [51], who observed that a combined 300 W US and 100 W MW technique further improved the solubility of myofibrillar protein (MP) by breaking down highly ordered aggregates into smaller filamentous and fragmented structures in exploring the influence of MW, US and combined ultrasound-microwave treatments at varying power levels on the structure and hydrolytic attributes of MP. However, Fig. 6 indicates that other combined US and MW treatments, including US100 + MW25, US50 + MW50, and US100 + MW50, along with MW50 alone, significantly decreased LPI solubility, showing a decline with escalating power—the highest power combination (US100 + MW50) resulting in the lowest solubility. This apparent contradiction illustrated that while mild US and MW combination enhanced solubility, excessive power (especially MW power) increased the degree of protein denaturation (as discussed in the secondary structure section), leading to the unfolding of proteins and exposing hydrophobic groups, promoting re-aggregation via hydrophobic interactions and consequently increasing particle size and decreasing solubility. Varghese and Pare [24] also observed a decrease in protein solubility as microwave power increased from 540 W to 810 W during microwave-assisted extraction of soy milk protein. They attributed this decline to the higher temperatures caused by increased microwave power, which promote protein denaturation, thereby exposing more hydrophobic amino acid residues in the soy milk, resulting in reduced protein solubility. This observation of reduced solubility with increased power has been found in different researches [52], [53].

In conclusion, E200 treatment, whether through US, MW, or their combination, significantly influences LPI solubility across various pH levels, with combined treatments offering a more efficient increase in solubility under optimal power settings. These findings suggest that combined US and MW treatments could be a promising approach to enhance the protein solubility of LPI. Further research is needed to refine these parameters for broader implementation in the food industry.

### 3.5 Particle size analysis

**Fig. 7:**
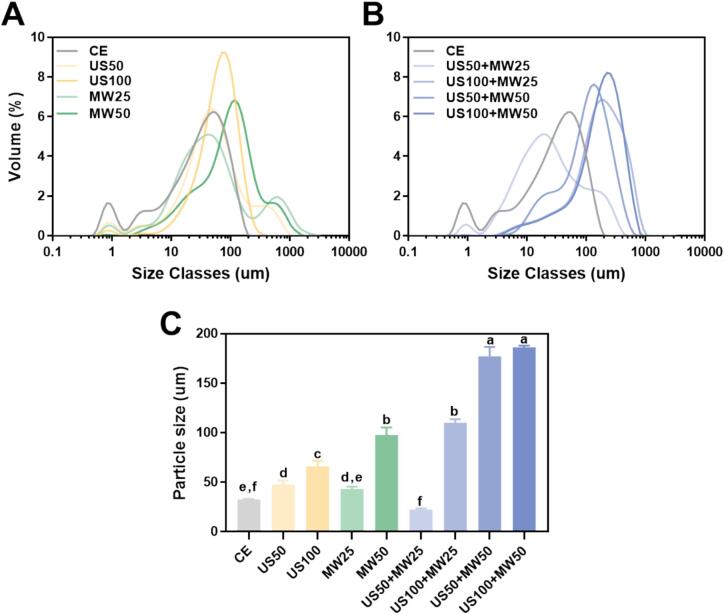
Particle size distribution of LPI processed from different parameters. A displayed the particle size distribution for CE, US, and MW used alone; B illustrated the particle size distribution for CE and the combination of US and MW; C provided the median particle size (D50) for all treatments.

This study explored the impact of various extraction methods on the particle size distribution of LPI with results presented in Fig. 7. Specifically, Fig. 7A displays the particle size distribution for CE, US, and MW used alone; Fig. 7B illustrates the particle size distribution for CE and the combination of US and MW; and Fig. 7C provides the median particle size (D50) for all treatments. The results indicated that both 10-minute treatments with US50 and MW25 effectively reduced the particle size of LPI, and were equivalent to the effect of CE for 1 h (Fig. 7A, C). This suggested that the cavitation effect of ultrasound or the thermal effects caused by microwaves might break hydrogen bonds and hydrophobic interactions and produce molecular unfolding, leading to a reduction in protein aggregates. This outcome aligned with findings from other studies investigating the use of ultrasound on plant proteins. For instance, Lo et al. [11] treated lupin protein suspensions at pH 5 and pH 9 with low-frequency ultrasound, significantly reducing particle sizes; similarly, Gao et al. [54] demonstrated that high-intensity ultrasound (HIUS) could effectively decrease the particle size of pea protein isolate (PPI).

However, when ultrasound power was elevated from 100 W to 200 W or microwave power from 500 W to 1000 W, the particle size of LPI correspondingly increased (Fig. 7A, C). This may be due to the rapid heating rates induced by intense ultrasound or microwave fields, which could accelerate protein molecule movement, raising the likelihood of collisions and leading to larger aggregates due to hydrophobic interactions. A similar result was observed by Li et al. [51], who discovered that the particle size of myofibrillar proteins (MP) became smaller under low-power microwave or ultrasonic treatment, but increased with further increase in microwave or ultrasonic intensity. Additionally, Zheng et al. [55] investigated the effect of microwave-assisted vacuum extraction on lotus seed protein, finding a trend of decreasing protein particle size at low to moderate power levels. However, as MW power increased, the particle size gradually increased. They suggested that lower power levels of MW treatment might partially break both intramolecular and intermolecular hydrogen bond structures, while the increase in protein particle size at higher powers was likely due to aggregate formation driven by intermolecular disulfide bridges and hydrophobic interactions.

Notably, the combination of ultrasound and microwave treatments synergistically optimized the extraction process. The combined 10-minute US50 + MW25 treatment resulted in the smallest particle size distribution (Fig. 7B, C), suggesting that the uniform heating effect caused by ultrasonic and microwave effectively destroyed the structure of proteins and promoted the cleavage of protein aggregates. But similarly, as the ultrasound and microwave power increased, the particle size of LPI would increase significantly, and the original bimodal distribution of CE and US50 + MW25 will become an obvious unimodal distribution with larger size (Fig. 7B), indicating that higher power levels in combined treatments tended to form larger LPI aggregates. The phenomenon of particle size initially decreasing and then increasing indicated that an appropriate combination of ultrasound and microwave treatment could reduce the aggregation of LPI, while excessive combined treatment may cause the re-aggregation of small-sized protein structures. Similarly, Rababah et al. [20] reported that nanoparticles from bitter lupin protein treated with the US + MW combination were twice the size of those treated with MW alone. Moreover, these changes in particle size corresponded with trends in LPI solubility measured after the treatments.

### 3.6 Viscosity analysis

**Fig. 8:**
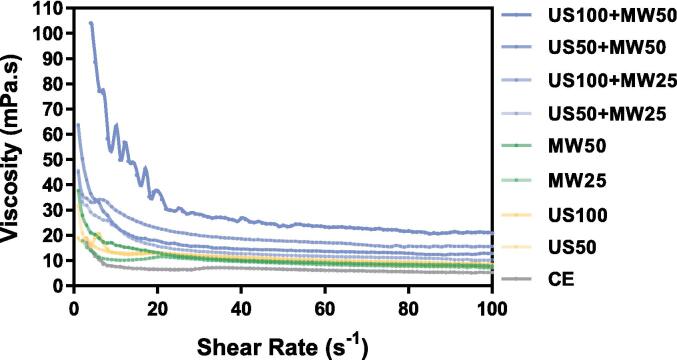
Viscosity-shear-rate of lupin protein isolate dispersions (15 % w/w), exposed to different parameters at pH 7 against the CE treatment.

Viscosity analysis of LPI showed a significant decrease in viscosity with an increasing shear rate for all treatments (Fig. 8), indicating typical shear-thinning behaviour of non-Newtonian fluids. Previous studies [3], [56] showed that the viscosity of isolated LPI exhibited shear-thinning behaviour, with the protein viscosity rapidly decreasing with increased shear forces. Additionally, separate US and MW treatments for 10 min resulted in low viscosity levels comparable to the 1-hour CE group, suggesting that both US and MW treatments could reduce the viscosity of LPI. Consistent with our findings, Lo et al. [11] reported that ultrasonic treatment reduced the viscosity of lupin suspensions at pH 9 from 25 Pa·s in the CE group to a minimum of 5.6 Pa·s. Also, in a study on the application of ultrasonic treatment to soy protein isolates (SPI) at neutral pH, Hu et al. [57] found that the apparent viscosity of the US-treated SPI was lower than that of the CE group. The possible explanation for the viscosity reduction is that US or MW treatment exposes more hydrophobic groups on the protein molecules' surface. O'sullivan et al. [58] claimed that the reduction in the intrinsic viscosity of US-treated isolated proteins indicated an increased level of hydrophobicity. In addition, Gharibzahedi and Smith [59], in their review, indicate that US treatment significantly reduces the intrinsic and bulk viscosities of legume protein solutions by exposing more hydrophobic groups on the protein molecules. This treatment induces dehydration and enhanced hydrophobicity, reducing the energy required for the proteins to adhere to the oil–water interface and decreasing the size and spacing of aggregates, further lowering the solution's viscosity.

The combined US50 + MW25 treatment showed lower viscosity; however, with increasing US and MW power, the combined US and MW treatment gradually increased the viscosity of LPI. This could be because, at lower power levels, the combination of US and MW treatment exposed more hydrophobic groups, leading to a decrease in viscosity, while a combination of higher power levels may cause small protein aggregates to form larger aggregates through hydrophobic interactions, reducing their hydrophobicity and increasing viscosity. The trend in lupin viscosity changes was consistent with the particle size and inversely correlated with solubility. These findings highlighted the potential of using US and MW-assisted methods to adjust the rheological properties of LPI to suit specific applications, although the highest power combination may increase viscosity.

## 4 Conclusions

In this study, we employed novel technique of combined US-MW treatment to extract and recover proteins from narrow-leaved lupin grain, variety ‘PRIMADONNA’, grown under the Irish temperate climate. The obtained LPI was analysed by protein recovery rates, and structural and functional analyses, including SDS-PAGE, FTIR, protein solubility, particle size, and viscosity analysis. The key innovation of this study lies in the use of combined US and MW technologies, which demonstrated a synergistic effect that significantly enhanced extraction efficiency compared to CE. This combined approach represents an advancement in plant protein extraction methods by leveraging the unique benefits of both technologies: the cavitation effect of US and the rapid, uniform heating of MW.

SDS-PAGE analysis showed that the primary structure of LPI did not change significantly, while FTIR revealed a trend of changes in the secondary structure. US treatment did not cause major protein denaturation; however, MW treatment at higher power (50 %, 1000 W) induced significant protein denaturation, transforming the protein structure from highly ordered (β-sheet and α-helix) to disordered structures (random coil and β-turn). The combined treatments (US50 + MW50, US100 + MW50) also exhibited these similar structural changes. Solubility analysis revealed that a lower power combination of US and MW (US50 + MW25) effectively increased protein solubility. In contrast, higher power combinations (US50 + MW50 and US100 + MW50) significantly reduced solubility due to severe protein denaturation, which exposed hydrophobic regions and formed insoluble aggregates. The trend in particle size changes was consistent with solubility, showing the smallest particle size in the US50 + MW25 treatment, but a significant increase with higher MW power. The viscosity curve correlated with solubility and particle size, with the highest viscosity observed in the US100 + MW50 treatment.

In summary, this study highlights the innovative potential of combined US and MW treatments for lupin protein extraction, offering a promising alternative to conventional methods. Future research could focus on optimizing the power combination so that this technology can maximize extraction yield and maintain protein functionality, making it highly adaptable for future applications in plant protein processing.

## CRediT authorship contribution statement

**Jiafei Tang:** Conceptualization, Investigation, Writing – original draft. **Lucie Cases:** Investigation, Resources, Writing – review & editing. **Sheila Alves:** Investigation, Resources, Writing – review & editing. **Da-Wen Sun:** Writing – review & editing, Supervision, Resources, Funding acquisition. **Brijesh K. Tiwari:** Supervision, Funding acquisition, Resources, Writing – review & editing.

## Declaration of competing interest

The authors declare that they have no known competing financial interests or personal relationships that could have appeared to influence the work reported in this paper.
